# Triterpene Composition and Bioactivities of *Centella asiatica*

**DOI:** 10.3390/molecules16021310

**Published:** 2011-01-28

**Authors:** Puziah Hashim, Hamidah Sidek, Mohd Helme M. Helan, Aidawati Sabery, Uma Devi Palanisamy, Mohd Ilham

**Affiliations:** 1 Halal Products Research Institute, Universiti Putra Malaysia, Putra Infoport, 43400 UPM Serdang, Selangor, Malaysia; 2 Industrial Biotechnology Research Centre, SIRIM Berhad, 1 Persiaran Dato’ Menteri, 40911 Shah Alam, Selangor, Malaysia; 3 School of Medicine and Health Sciences, Monash University Sunway Campus, Jalan Lagoon Selatan, 46150 Bandar Sunway, Selangor, Malaysia; 4 Forest Research Institute Malaysia, 52109, Kepong, Selangor, Malaysia

**Keywords:** *Centella asiatica*, triterpenes, collagen, anti-oxidant, UV protection

## Abstract

Leaves of *Centella asiatica* (Centella) were analysed for their triterpene composition and bioactivity such as collagen enhancement, antioxidant, anticellulite and UV protection capacity properties. Triterpenes of Centella were measured using HPLC-PAD on an Excil ODS 5 μm (C18) column for the simultaneous determination of asiatic acid, madecassic acid, asiaticoside and madecassoside. Centella was found to contain significant amounts of madecassoside (3.10 ± 4.58 mg/mL) and asiaticoside (1.97 ± 2.65 mg/mL), but was low in asiatic and madecassic acid. The highest collagen synthesis was found at 50 mg/mL of Centella extracts. The antioxidant activity of Centella (84%) was compared to grape seed extract (83%) and Vitamin C (88%). Its lipolytic activity was observed by the release of glycerol (115.9 µmol/L) at 0.02% concentration. Centella extracts exhibited similar UV protection effect to OMC at 10% concentration. In view of these results, the potential application of Centella in food and pharmaceutical industries is now widely open.

## 1. Introduction

*Centella asiatica* (L) Urban (Umbelliferae) has been used as a traditional herbal medicine in Malaysia and other parts of Asia for hundreds of years [1]. It is commonly known as pegaga in Malaysia, pennywort and gotu kola in America. Besides its common use as a medicinal plant, it is also eaten fresh as salad, cooked as vegetable and blended as a drink. It is also used in nutraceutical preparations, thus becoming an important commercial plant [2]. This tropical plant has been reported to have been used for various medicinal purposes such as for wound healing [3,4,5], treatment of asthma, ulcers, lepsory, lupus, vein diseases [1,6], memory improvement [7,8], as an antidepressant [9], antibacterial, antifungal [10], psoriasis [11] and anti-cancer agent [12] even though its primary application has been in promoting wound healing.

Based on the numerous studies, the biologically active ingredients are believed to be its triterpenes [13], and the medicinal values of this plant are mainly attributed to the presence of several triterpenes, namely asiatic acid, madecassic acid, asiaticoside and madecassoside [14,15]. Triterpenes being the major components of Centella, they have been regarded as its biomarker components [16]. Quantification of triterpenes of Centella has been successfully established by several researchers using HPLC-UV [17,18,19,20,21,22], however, the triterpene components in Centella are known to be vary depending on its growth location and the diverse environmental conditions it is subjected to [2,6]. 

Cell culture studies have shown that asiatic acid was the only triterpene component responsible for stimulating collagen synthesis of human fibroblasts [23]. Other studies have shown that the extract from Centella containing three triterpenic constitutents, *i.e.,* asiatic acid, madecassic acid and asiaticoside, and their mixtures were able to stimulate collagen synthesis in skin fibroblast culture [4], while asiaticoside and madecassoside of the plant stimulated type-I collagen and stimulated type-III collagen, respectively [24]. Titrated extract of Centella which contained the same three triterpenes asiatic acid, madecassic acid and asiaticoside, promoted both fibronectin and collagen synthesis by 20-35% [3]. In an experimental animal model, the triterpenes of Centella have been shown to stimulate collagen and glycosaminoglycan synthesis [5]. 

Due to its ability to stimulate collagen, Centella has been used in skin care products for restoring skin firmness, elasticity and improving skin appearance [25]. Topical formulations of the extract when applied on experimental wound treated rats’ epitheliased faster and the healing rate of contraction increased [26]. In addition, Centella extract improved the micro-circulation effect and capillary permeability in patients with venous hypertension [27]. The extract was also seen to affect lipolytic activity, resulting in the increase of cyclic adenosine monophosphate (cAMP) content in the human adipocytes, causing a slimming effect in humans [28]. 

The antioxidative property of Centella may play an important role in reducing the activity of reactive oxygen species (ROS) in the body system [29,30]. Its antioxidant capacity proved to be neuroprotective and able to protect rat brain against age related oxidative damage [31]. Extracts from Centella have been reported by many researchers to exhibit antioxidant properties [9,29,32]. It has been reported that induction of antioxidant level in wound healing is due to asiaticoside [34] and flavonoids [30]. There are no reports on the ability of Centella extracts to provide UV protection. The aim of this study is to evaluate the triterpene composition of Centella, and its bioactivity towards enhancement of collagen, antioxidant, lipolytic and UV protection properties. 

## 2. Results and Discussion 

The HPLC gradient method suing a PDA detector at 205 nm is able to simultaneously quantify four triterpenes in Centella extract, which are madecassoside, asiaticoside, madecassic acid and asiatic acid. Several researchers were able to detect these triterpenes at a wavelength of 220 nm using a gradient system of different solvents – water and acetonitrile [17,20,22]. 

**Figure 1:**
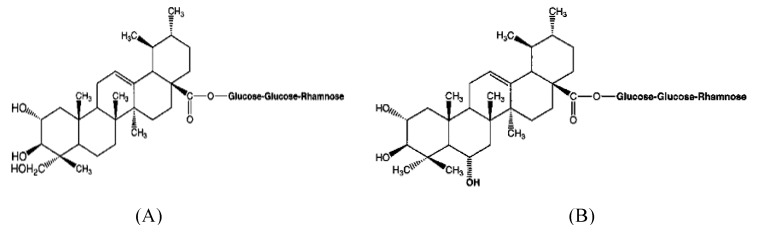
Triterpenes of Centella.

Table 1 shows that the extract of Centella leaves comprised the four triterpenes. This extract contains the highest amount of glycosides than the aglyconic content. Madecassoside is the highest component, followed by asiaticoside at 3.10 ± 4.58 and 1.97 ± 2.65 mg/mL, respectively. Both asiatic and madecassic acid are found at 0.55 ± 2.29 and 0.55 ± 0.89 mg/mL, respectively. However, studies carried out by Zainol *et al*. [35] using an isocratic system of methanol and water in a ratio of 8:2 was able to separate three triterpenes (asiaticoside, madecassoside and asiatic asid) in the leaves of Centella acessions collected from the southern part of Malaysia with a high concentration of asiatic acid (3.4 mg/mL), whereas those of asiaticoside and madecassoside were 2.6 mg/mL and 5.3 mg/mL, respectively. The triterpene components in Centella are not always the same due to the location of the plant and diverse environmental conditions [2,6] and it also due to different accessions [21]. Figure 1 shows the structures of these triterpenes. The difference between the glycosides and aglycones (asiatic and madecassic acids) is the sugar glucose-glucose-rhamnose, whereby for the aglycones, the sugar molecule is replaced by an OH group. 

**Figure 2:**
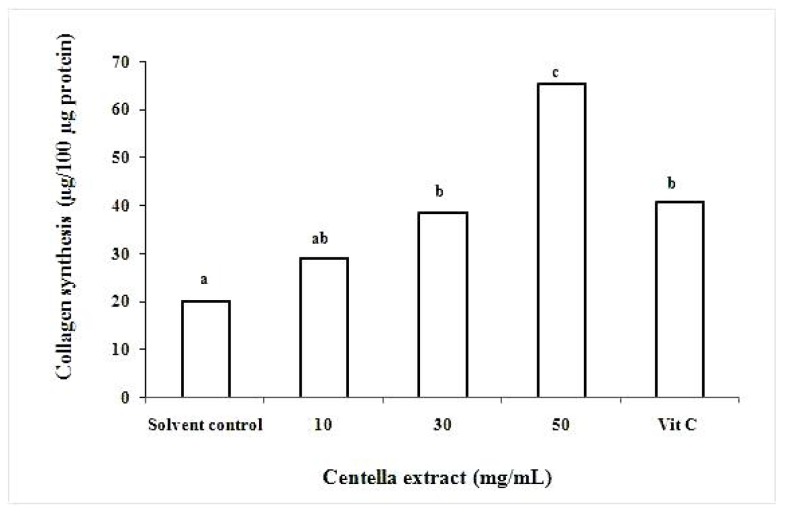
Stimulation of collagen synthesis of fibroblast cells using different concentration of Centella extracts (mg/mL) and vitamin C (25 µg/mL) was used as positive control. Untreated solvent was used as control. All fibroblast were incubated at 5% CO_2_ for 48 h incubation. Values are the mean ± SD (n = 3). Values with different letter are significant different at *P < 0.05*.

Centella extract showed stimulatory effects on collagen synthesis in a dose dependent manner (Figure 2). The data was expressed as µg of collagen synthesized per 100 µg of total cellular protein in order to standardize the total number of cells from each plate. The vitamin C (25 µg/mL) which was used as a positive control showed 2-fold collagen enhancing response. At 50 mg/mL, the Centella extract enhanced 3-fold collagen production compared to the control (untreated), whereas at 30 mg/mL and 10 mg/mL the collagen enhancement were of 2- and 1.4-fold, respectively. Centella extract at higher concentration than 50 mg/mL, the cell viability was decreased (data not shown). The results obtained are in agreement with previous studies whereby the triterpenes extracted from Centella stimulated collagen synthesis [4,5]. The stimulation of collagen synthesis is important criteria for plant extract to be a potential ingredient in skin care products for restoring skin firmness, elasticity and improving skin appearance [25].

**Figure 3:**
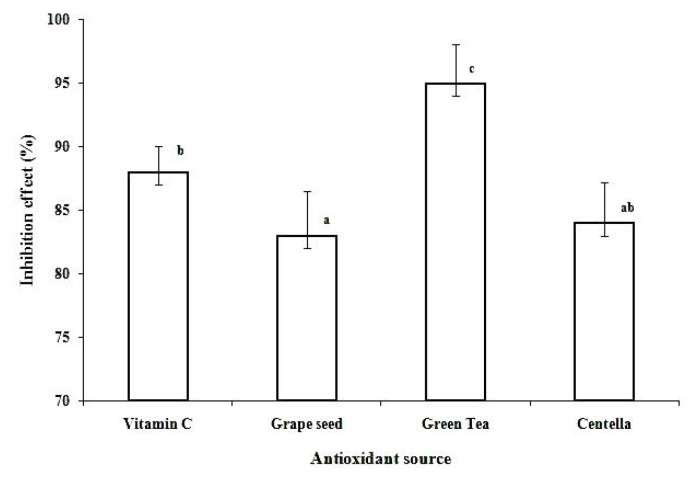
DPPH-radical scavenging activity of centella extract, grape seed, green tea and vitamin C at 1 mg/mL concentrations. Values are the mean ± SD (n = 3). Among the extracts studies, green tea shows the highest inhibition effect followed by vitamin C, Centella and grape seed extract. Values with different letter are significantly different at *P < 0.05*.

The antioxidant activity of Centella extract was evaluated by measuring its ability to scavenge DPPH free radicals while vitamin C, green tea and grape seed extract (1 mg/mL each) were used as positive controls. As shown in Figure 3, Centella extract demonstrated profound free radical-scavenging activity of 83% inhibition at a concentration of 1 mg/mL. This finding corroborates with similar work reported in several studies [9,29,32]. It was found that the inhibition effect of the free radical scavenging activity decreased in the order of green tea > vitamin C > Centella > grape seed extract. Centella extract exhibited comparable activity with grape seed extract which is claimed to be a powerful antioxidant due to its proanthocyanidin content [36,37]. Asiaticoside, which is present in a high quantity (1.97 mg/mL), may be a significant contributor to the observed antioxidant activity of Centella. Shukla *et al*. [34] have in fact reported that the enhancement of antioxidant activity might due to both Centella’s asiaticoside and flavonoid content [30]. Our findings also agree with the previous studies by Hamid *et al*. [29], who has reported that antioxidant activity of Centella was highest in its ethanol extract. Ling *et al.* [38] in their antioxidant study of Malaysian plants have also reported similar superior scavenging activity in ethanolic extracts and have linked it to its raised total phenolic content.

**Figure 4:**
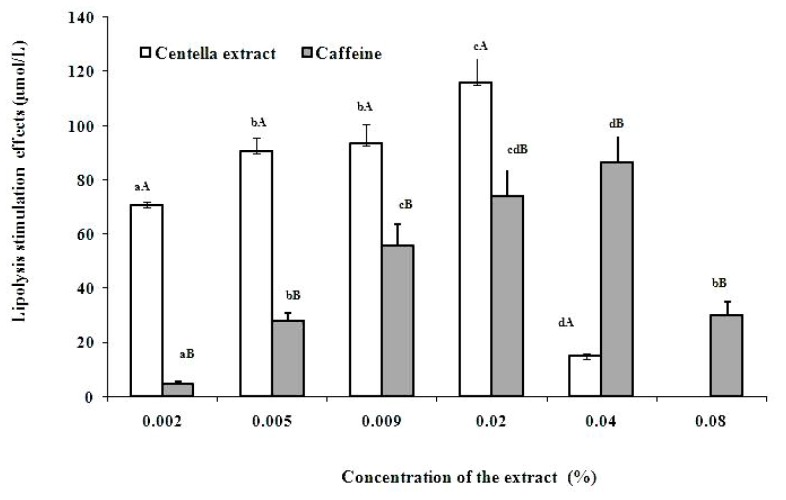
Effect of Centella extract and caffeine on lipolysis activity of adipocytes. Caffeine (1 mg/mL) was used as positive control and untreated solvent was used as control. The adipocytes with the positive control and test samples were incubated for 30 min at 37 °C with constant shaking in a CO_2_ incubator. Then infranatant was heated at 70 °C for 10 min and glycerol was measured using Randox kit. Values are the mean ± SD (n = 3).

The ability of Centella extract to stimulate lipolysis in adipocytes in a dose dependent manner is shown in Figure 4. The Centella extract has the highest release of glycerol compared to the positive control caffeine at concentration from 0.002% to 0.02%. 

The maximum glycerol release of 115.9 µmol/L was recorded at 0.02% concentration of the extract. In contrast, at the same concentration, the positive control, caffeine only exhibited a glycerol release of 74.0 µmol/L. Furthermore, the extracts showed activity even at low concentrations of 0.002% but no activity was recorded above a concentration of 0.08%. However, it was noted that the positive control caffeine commonly used in anti-cellulite treatment only show better activity at concentration 0.04%. This results support the claimed that Centella extract improve the micro-circulation effect and capillary permeability in patients with venous hypertension [27]. The extract was shown the affect lipolytic activity, resulting in the increase of cyclic adenosine monophospate (cAMP) content in the human adipocytes which can assist in slimming effect in human [28]. They have demonstrated that the Centella extract is one of the effective micro-circulation activators present in the slimming liposomes. 

**Figure 5:**
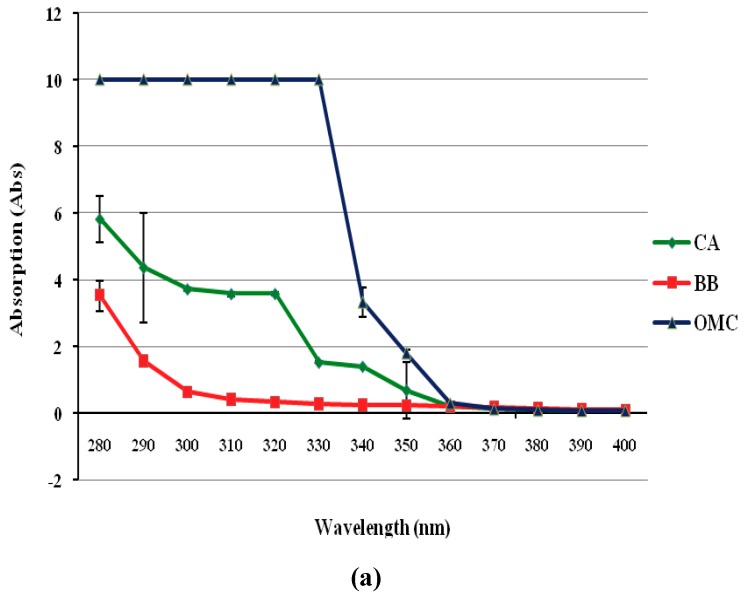

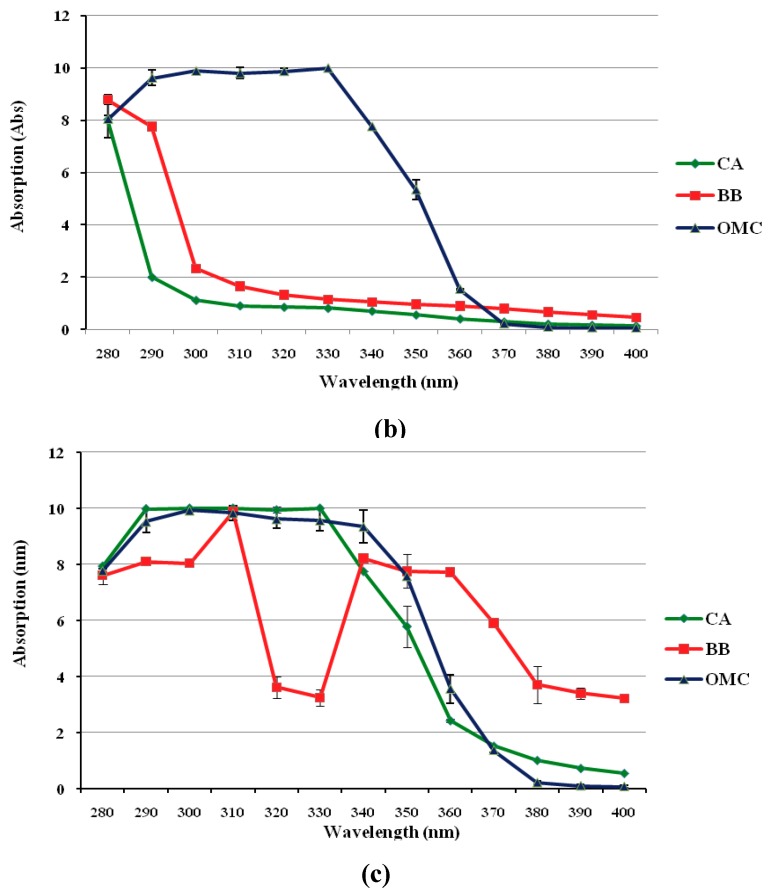
The protection effect of Centella extract (CA), bearberry extract (BB) and octyl methoxy cinnamate (OMC) against UVA (ultraviolet-A) and UVB (ultraviolet-B) damage at different concentration; (a) at 0.1%, (b) at 1.0% and (c) at 10.0%. The 10% Centella extract shows comparable results with OMC.

The protective effect of Centella extract at various concentration (0.1, 1.0 and 10.0%) against UVA (ultraviolet-A) and UVB (ultraviolet-B) absorbance is presented in Figure 5. The UV protection effect was compared to positive controls (OMC and bearberry extract). At the same concentration (0.1 and 1.0%), it appears that the Centella extract UV absorption ability against UVB (290-320 nm) is mild compared to OMC. Only at a higher concentration (10.0%) the Centella extract demonstrated higher absorbance and was comparable with OMC in its protection against UVB absorbance. This result also showed that the Centella extract is better than the bearberry extract at 10% concentration. The OMC was used in this experiment as it is the most frequently-used chemical sunscreen-active ingredient today, preventing skin damage, is a good UVB filter and is less potent [39]. 

Bearberry extract was selected as the positive control because it contains arbutin, which has whitening effects and UV protection capabilities [40]. The UVB is a short-wave solar of 290-320 nm, it is more powerful than UVA in producing sunburn and may cause basal and squamous cell carcinomas as well as melanomas [41]. Whereas UVA is long-wave solar rays of 320-400 nm, less likely to cause sunburn but it penetrate the skin to cause skin wrinkle and photo-aging. The UV protection ability shown by Centella extract might be due to its antioxidant capability and its triterpene(s) components. Many cases the antioxidant properties do assist in photostabilizing capabilities on UV filter protection [38]. The triterpenes from *Panax Notoginseng* plant from China also demonstrated similar finding on UV protection activity [42].

## 3. Experimental 

### 3.1. Plant materials

The Centella plant was obtained from the Forest Research Institute Malaysia (FRIM), Kepong, Selangor, Malaysia. The herbarium specimen of Centella (FRI50032) was deposited at the Medicinal Plant Division, FRIM. 

### 3.2. Chemicals and materials

The chemical standards of asiatic acid, madecassic acid, asiaticoside and madecassoside were purchased from Extrasynthese (Genay, France). HPLC grade phosphoric acid and acetonitrile were supplied by Fisher Scientific (Pittsburgh, PA). Sirius red, collagen type-I, L-glutamine and penicillin-streptomycin antibiotics were obtained from Sigma Aldrich (St. Louis, USA). Tissue culture media (DMEM) and heat inactivated fetal bovine serum were purchased from Difco (Detroit, Michigan, USA). Human foreskin fibroblast cells CCD-1114Sk (CRL 2450) was from ATCC (American Type Culture Collection, Manassas, VA, USA). All other reagents used were of analytical grade.

### 3.3. Preparation of ethanolic-aqueous extraction

The aerial part of Centella was cut and dried in oven at 50 °C for 3-7 days. Dried samples were grounded into powder form using a mini grinder (1.5 mm). Powdered samples (18 g) were refluxed with 36% ethanol (approximately 108 mL) at a ratio of 1:6 for 1 h at 60 °C. Then, the extract was filtered through Whatman filter paper and 60 mL of extract obtained was mixed with propylene glycol (80% in water, 40 mL) and stored at 4 °C. 

### 3.4. High performance liquid chromatography (HPLC) analysis 

Triterpene standards were prepared in four different concentrations. A stock solution (800 μg/mL) of asiatic acid and madecassic acid were diluted with HPLC grade methanol to obtain a concentration of 40, 20, 10 and 5 µg/mL, respectively. A stock solution (800 μg/mL) of madecassoside and asiaticoside were diluted to obtain a concentration of 120, 60, 30 and 15 µg/mL.

HPLC analysis was carried out with a Waters HPLC system, comprising of Waters 600E System Controller, WATERS 996 photodiode array (PDA) detector, a personal computer with Empower software and Rheodyne injector. The column used was an Excil ODS 5 μm (C18), (150 mm × 4.6 mm). The detection wavelength was set at 205 nm. The mobile phase used for the separation was 0.05% phosphoric acid (solvent A) and 100% acetonitrile (solvent B) at a flow rate of 1 mL/min. 

The samples were dissolved in methanol/ethanol-aqueous to make up a concentration of 1 mg/mL. Prior to injection, samples and standards were filtered through a 0.45 μm nylon membrane syringe filter. The injection volume was 20 μL with three injections being performed for each samples and standards. The concentrations of triterpenes in the samples were estimated from the standard curve that has been prepared by plotting peak area against concentration of standards: asiatic acid, madecassic acid, asiaticoside and madecassoside with coefficient (r^2^) greater than 0.99. 

### 3.5. Protein assay

Protein concentration of the cell lysate produced with acetic acid buffer was determined by the Coomassie blue dye binding method using the dye concentrates from Bio-Rad and bovine serum albumin as protein standard.

### 3.6. Culture of human dermal fibroblasts preparation

The human dermal fibroblast cells (ATCC CRL-2450) were cultured in Dulbecco’s modified Eagle’s medium (DMEM) with 10% fetal bovine serum, 2 mM L-glutamine, 100 IU/mL penicillin and 100 µg/mL streptomycin antibiotics. The cells were seeded on a 25 cm^2^ flask (1 × 10^5^ cells/mL) for 48 h in a 5% CO_2_ incubator at 37 °C. The confluent fibroblast cells were treated with various concentrations of Centella extract and vitamin C (25 µg/mL) was used as positive control and subjected for 48 h incubation. The treated cells were harvested, followed by extraction with 0.5 N acetic acid (pH 2.0) and addition of protease inhibitor cocktail. Then, it was sonicated for 15 s at 15,000 Hz to solubilize the cell-bound collagen and the cell lysate was quantitated using a sirius red staining method. 

### 3.7. Assay of Collagen by sirius red staining method

Human collagen Type-I at 200 µg/mL was used as the standard and 0.05 M acetic acid as the blank. A sample (50 µL), collagen standard, solvent control (propylene glycol 80% in water) and blank were added to the appropriate wells in a multiscreen plate, followed by sirius red solution (250 µL) to each well and mixed at room temperature for 30 min to allow precipitation. The excess solution was removed under vacuum for each wash. Then 0.5 M NaOH (250 µL) was added into each well and mixed at room temperature to resuspend the precipitate. Aliquots (100 µL) were transferred to a clear 96 well plate and absorbance was measured at 540 nm. The amount of collagen is expresses as micrograms per 100 µg protein. 

### 3.8. Lipolytic activity

The cultured fibroblast cells grown to confluence in DMEM were stimulated to differentiate by addition of fresh medium containing 0.5 mM IBMX (3-isobutylmethylxanthine), 0.1 µM dexamethasone and 1.7 µM insulin for 3 days followed by treatment only with 1.7 µM insulin. Differentiation was complete after 12-15 days of treatment. One day before the experiment, medium was replaced with fresh medium lacking insulin. To measure lipolysis, fat cells were placed in plastic vials/wells (1 × 10^5^ cells/mL) in Krebs-Ringer bicarbonate 30 mM Hepes, 1% BSA, 2.5 mM glucose as the incubation buffer at pH 7.4. Adenosine deaminase (10 ug/mL) is added to the incubation medium to prevent the accumulation of adenosine which inhibits lipolysis. Positive control (caffeic acid, 1 mg/mL) and test samples are added to the cells and incubated for 30 min/h at 37 °C with constant shaking in a CO_2_ incubator. At the end of the incubation, infranatant was removed (after standing for 5 min) and heated at 70 °C for 10 min to inactivate and enzymes released by the cells. The infranatant was assayed for glycerol release using the Randox glycerol kit. The results were compared with basal lipolysis of the fat cells which is the cells that were not treated with any sample.

### 3.9. Measurement of the free radical scavenging activity

The scavenging effect of extracts on 2,2-diphenyl-1-picrylhydrazyl (DPPH) radical was estimated as described by Lee *et al*. (2003). Extracts (1 mg/mL) was mixed with 0.5 mM DPPH in ethanol solution (0.25 mL) and 100 mM acetate buffer (0.5 mL, pH 5.5). The mixture was shaken and left to stand at room temperature for 30 min before the absorbance of the resulting solution was measured spectrophotometrically at 517 nm. The negative control used was DPPH solution in ethanol (950 µL) with ethanol (50 µL) while the positive controls (1 mg/mL) used were commercially available grape seed (Vitis vital^TM^), vitamin C and green tea. Scavenging activity in this assay was expressed as % inhibition of the free radical scavenging activity compared to the negative control.

### 3.10. UV protection activity

The Centella extract at different concentrations were dissolved in ethanol and screened with UV-Vis spectrophotometric through wavelength of 280-400 nm. Similar screening was conducted with ethanol solvent as the negative control, whereas octyl methoxy cinnamate (OMC) and bearberry extract were used as the positive control.

### 3.11. Statistical analysis

Experiments were carried out in triplicate at (*P < 0.05*) and statistical analysis was accomplished with SAS software, using Duncan’s multiple comparison test. 

## 4. Conclusions

Centella extract exhibited several potential bioactivities which could be of potential commercial interest in the food and pharmaceutical industries. Evaluation on Centella has found its chemical composition consist of four triterpenes, namely madecassoside, asiaticoside, madecassic acid and asiatic acid. The extract significantly stimulated collagen synthesis, better than vitamin C, and the lipolysis activity was found to be better than that of caffeine. The extract also showed an inhibitory effect with regards to free radical scavenging activity, comparable to grape seed extract. This preliminary UV study suggested that the Centella extract could be a potential natural protection against UVB damage and this activity might be due to its triterpene component(s). Therefore, further experiments are required to identify the component(s) responsible. 

## Figures and Tables

A: Asiaticoside and asiatic acid (without Glucose-Glucose-Rhamnose); B: Madecassoside and madecassic acid (without Glucose-Glucose-Rhamnose).

(a-d): Values with diﬀerent letters are signiﬁcantly diﬀerent at *P* < 0.05 for the same source of extract; (A-B): Values with different letters are significantly different at *P* < 0.05 for each concentration of the extract.

**Table 1:** Triterpene concentration of Centella extract.

Types of triterpenes	Retention time (min)	Concentration of triterpenes in Centella extract (mg/mL)
Madecassoside	6.313	3.10 ± 4.58^a^
Asiaticoside	7.997	1.97 ± 2.65^b^
Madecassic acid	12.199	0.55 ± 2.29^c^
Asiatic acid	13.573	0.55 ± 0.89^c^
